# Silver-Assisted Chemical Etching for the Fabrication of Porous Silicon N-Doped Nanohollow Carbon Spheres Composite Anodes to Enhance Electrochemical Performance

**DOI:** 10.3390/ma17133189

**Published:** 2024-06-29

**Authors:** Zimu Zhang, Yuqi Zhang, Weixuan Chen, Xiang Zhang, Le Yu, Zisheng Guan

**Affiliations:** College of Materials Science and Engineering, Nanjing Tech University, 30 South PuZhu Road, Nanjing 210009, China; 202161203162@njtech.edu.cn (Z.Z.); 202161203205@njtech.edu.cn (Y.Z.); 202161203185@njtech.edu.cn (W.C.); 202161203285@njtech.edu.cn (X.Z.); 202261203158@njtech.edu.cn (L.Y.)

**Keywords:** silver-assisted chemical etching, porous silicon, silicon/carbon composite anodes, lithium-ion batteries

## Abstract

Silicon (Si) shows great potential as an anode material for lithium-ion batteries. However, it experiences significant expansion in volume as it undergoes the charging and discharging cycles, presenting challenges for practical implementation. Nanostructured Si has emerged as a viable solution to address these challenges. However, it requires a complex preparation process and high costs. In order to explore the above problems, this study devised an innovative approach to create Si/C composite anodes: micron-porous silicon (p-Si) was synthesized at low cost at a lower silver ion concentration, and then porous silicon-coated carbon (p-Si@C) composites were prepared by compositing nanohollow carbon spheres with porous silicon, which had good electrochemical properties. The initial coulombic efficiency of the composite was 76.51%. After undergoing 250 cycles at a current density of 0.2 A·g^−1^, the composites exhibited a capacity of 1008.84 mAh·g^−1^. Even when subjected to a current density of 1 A·g^−1^, the composites sustained a discharge capacity of 485.93 mAh·g^−1^ even after completing 1000 cycles. The employment of micron-structured p-Si improves cycling stability, which is primarily due to the porous space it provides. This porous structure helps alleviate the mechanical stress caused by volume expansion and prevents Si particles from detaching from the electrodes. The increased surface area facilitates a longer pathway for lithium-ion transport, thereby encouraging a more even distribution of lithium ions and mitigating the localized expansion of Si particles during cycling. Additionally, when Si particles expand, the hollow carbon nanospheres are capable of absorbing the resulting stress, thus preventing the electrode from cracking. The as-prepared p-Si utilizing metal-assisted chemical etching holds promising prospects as an anode material for lithium-ion batteries.

## 1. Introduction

Lithium-ion batteries (LIBs) play a crucial role as a rechargeable battery technology widely used in electric vehicles, portable electronics, and energy storage systems. This is due to their extended cycle life, high energy density, and environmentally friendly nature. [1,2,3,4,5]. Commercial anode materials mainly rely on graphite, boasting a theoretical specific capacity of 372 mAh·g^−1^ [6,7,8]. However, their limited capacity falls short of meeting the evolving demands for the advancement of LIBs. Hence, there is a pressing need to explore alternative anode materials to augment the specific capacity LIBs. Si emerges as an appealing candidate for anode material with its high theoretical capacity of 4200 mAh·g^−1^ and a lower lithiation potential (~0.5 V vs. Li/Li^+^) [9,10,11]. Moreover, Si is second only to oxygen in terms of reserves in crust, significantly reducing the raw material costs [12,13]. However, the substantial volume expansion during lithiation results in particle crushing, electrode cracking, and solid electrolyte interface (SEI) films that continue to accumulate [14,15,16]. Additionally, being a semiconductor material, Si possesses a significant energy gap that imposes limitations on electron conductivity. As anode material, these drawbacks present substantial challenges for commercial applications [17].

Researchers extensively explore solutions to overcome the aforementioned challenges. Huang et al. observed a critical particle size for Si nanoparticles during the initial lithiation process. When the particle size of Si particles is below 150 nanometers, the structure can remain stable [18]. Therefore, nanometer-sized Si, encompassing nanoparticles [19,20], nanowires [21,22], nanorods [23,24], nanotubes [25,26], and nanosheets, [27,28] can effectively mitigate volume expansion. However, the high production costs currently hinder the widespread commercial application of the nanometer-sized Si [29,30]. Designing Si with specific structures, such as sandwich, yolk–shell, and core–shell structures is also an effective strategy [31,32,33]. Approaches like carbon coating, doping, and constructing three-dimensional conductive networks are commonly adopted to improve the conductivity of Si-based composites [34,35,36].

In recent years, the porous structures have garnered increasing attention due to their abundant pores, which can alleviate the mechanical stresses of volume expansion. Various methods exist for as-prepared p-Si. Xu et al. utilized P and Sr-doped Al-Si alloys for the p-Si preparation through dealloying, after undergoing 100 cycles, achieving a discharge capacity of 110 mAh·g^−1^ at a current density of 0.2 A·g^−1^ [37]. Wang et al. obtained p-Si particles using the magnesium thermoreduction method, etching Si mud waste from photovoltaic cutting, and after undergoing 100 cycles, achieved a discharge capacity of 751.1 mAh·g^−1^ at a current density of 0.1 A·g^−1^ [38]. However, these methods involve complex equipment and operations, hindering the mass production of stable and advanced performance Si-based anode composites for LIBs. As a top–down method for p-Si preparation, metal-assisted chemical etching offers simplicity and low-cost advantages, making it an ideal choice for the mass production of p-Si [39,40].

In this study, the p-Si was prepared by silver-assisted chemical etching. There has been extensive reporting on the silver-assisted chemical etching of p-Si [7,41,42,43,44]. Notably, in this work, the p-Si materials with abundant pores were prepared using an ultra-low concentration of silver nitrate (6 mM) solution, thus significantly reducing the production cost and achieving yields of more than 55%. Subsequently, we prepared the p-Si@C composites connected with hollow carbon nanospheres, utilizing SiO_2_ as a template and pyrolyzing PDA and etching the SiO_2_ by HF. The three-dimensional network formed by the hollow carbon nanospheres has the capability to absorb stress resulting from the expansion of Si particles, effectively averting electrode cracking. This network expedites electronic transmission, promotes a uniform dispersion of lithium ions, and alleviates the adverse effects of Si volume expansion on the electrode structure, thereby prolonging the cycle life of the battery. Furthermore, the electrochemical properties of the p-Si particles obtained through etching surpass those of pure Si particles.

## 2. Materials and Methods

### 2.1. Material Preparation

The as-prepared p-Si underwent the following steps: 8 g of commercially available Si powder (about 5 μm, 99.99%) was added to a mixed solution of 2 mL 10 M H_2_O_2_ (purchased from Lingfeng Chemical Reagent Co., Ltd., Shanghai, China), 0.1 mL 0.25 M NaOH (purchased from Xilong Scientific Co., Ltd., Guangdong, China) and 198 mL deionized water and dispersed by ultrasonication for 5 min, filtration of the Si powder and washing with deionized water several times. The filtered Si powder was then added into a mixed solution of 4 mL 22.6 M HF (purchased from Sinopharm Chemical Reagent Co., Ltd., Shanghai, China), 0.4 mL 6 mM AgNO_3_ (purchased from Sinopharm Chemical Reagent Co., Ltd., Shanghai, China) and 395.6 mL deionized water and slowly stirred for 5 min. Filter out the silver-plated Si powder and rinse the residual reagent with deionized water. The as-prepared Si powder was added into a mixed solution of 20 mL 10 M H_2_O_2_, 48 mL 22.6 M HF, 2 mL of auxiliary additives (purchased from Nanjing Naxin New Materials Co., Ltd., Nanjing, China) and 370 mL deionized water, and then it reacted at 60 °C for 1 h. The resulting p-Si was obtained after several washes with deionized water, filtration, and drying.

The synthesis of p-Si@C involved the following sequential steps. Initially, 0.2 g of the p-Si was dispersed ultrasonically in a mixed solution comprising 40 mL of absolute alcohol, 10 mL of deionized water, and 4 mL of ammonia water (purchased from Lingfeng Chemical Reagent Co., Ltd., Shanghai, China). To this dispersion, 2 mL of tetraethyl orthosilicate (TEOS) (purchased from Sinopharm Chemical Reagent Co., Ltd., Shanghai, China) was added, and the solution was stirred for 12 h at room temperature. The resulting p-Si@SiO_2_ particles were collected through filtration, subjected to multiple washes with deionized water, and dried. Then, 0.5 g of the p-Si@SiO_2_ was ultrasonically dispersed in 100 mL Tris buffer (10 mM, pH 8.5) (purchased from Phygene, Fuzhou, China). To this dispersion, 0.5 g of dopamine hydrochloride (DA) (purchased from Macklin Biochemical Co., Ltd., Shanghai, China) was added, and the solution was stirred for 24 h at room temperature. After washing with deionized water and drying, the desired product was obtained by annealing in a tube furnace under an argon atmosphere at 800 °C for 3 h, following a heating rate of 3 °C min^−1^, resulting in p-Si@SiO_2_@C. Finally, the p-Si@SiO_2_@C was introduced into a water solution containing 10% HF and stirred for 30 min. Following multiple washes with deionized water, filtration, and drying, the p-Si@C composites interconnected by a carbon shell network were obtained. Si@C was synthesized using a similar method, omitting the etching step, compared to the p-Si@C.

### 2.2. Material Characterization

A scanning electron microscope (SEM, FEI Scios 2 HiVac, Thermo Fisher Scientific, Waltham, MA, USA) equipped with an energy-dispersive X-ray spectrometer (EDS), as well as a transmission electron microscope (TEM, FEI Talos F200X G2, Thermo Fisher Scientific, Waltham, MA, USA), were employed to characterize the structure and elemental distribution of the composites. The specific surface area and the pore size distribution were analyzed by Brunauer–Emmett–Teller (BET, ASAP 2460, Micromeritics, Norcross, GA, USA) measurements with a Micrometrics ASAP 2460 surface area analyzer. For qualitative analysis of the as-prepared samples, we used X-ray diffraction (XRD, SmartLa, Rigaku Corporation, Akishima-shi, Japan). The weight loss process of composites analyses is conducted on the thermogravimetric analysis (TGA, Netzsch STA 449 F3, Selb, Germany) at a heating rate of 10 °C min^−1^ from 30 to 800 °C in the Ar atmosphere. For the structural characterization of carbon in composites, we used Raman spectroscopy (InVia Reflex, Renishaw, New Mills, UK). In addition, we also employed X-ray photoelectron spectroscopy (XPS, K-Alpha, Thermo Scientific, Waltham, MA, USA) to examine the elemental composition and valence states of the composite surface.

### 2.3. Electrochemical Measurements

The active materials, conductive agent acetylene black (purchased from Zhuguang New Energy Technology Co., Ltd., Guangdong, China), and binder carboxymethyl cellulose (CMC) (purchased from 3A Chemicals, Anqing, China) were thoroughly mixed in a deionized water solution with a weight ratio of 7:2:1 to form a slurry. We scraped the slurry onto a current collector, and the resulting material was dried at 100 °C under vacuum for 12 h. Subsequently, electrodes were punched into 12 mm diameter discs with an active substance loading of 0.5 mg·cm^−2^. These electrodes were assembled into 2032-type coin cells within an Ar-filled glove box. Celgard 2500 served as the separator, a lithium chip functioned as the reference electrode, and the electrolyte comprised 1 M LiPF6 in ethylene carbonate (EC)/ethyl methyl carbonate (EMC) (1:1 by volume), including 5% fluoroethylene carbonate (FEC). Galvanostatic charge–discharge performance testing was conducted using a NEW-WARE battery tester (purchased from NEWARE Electronics Co., Ltd., Shenzhen, China) within potential windows of 0.01–3 V. Cyclic voltammetry (CV) and electrochemical impedance spectroscopy (EIS) were performed on a CHI660 electrochemical analyzer (purchased from Wuhan LAND Electronic Co., Ltd., Wuhan, China). The CV voltage range was 0.01–1.50 V (vs. Li/Li^+^) with a scan rate of 0.10 mV·s^−1^, while the EIS test was conducted in the frequency range of 0.01–100 kHz with an amplitude of 5 mV.

## 3. Results and Discussion

**Figure 1:**
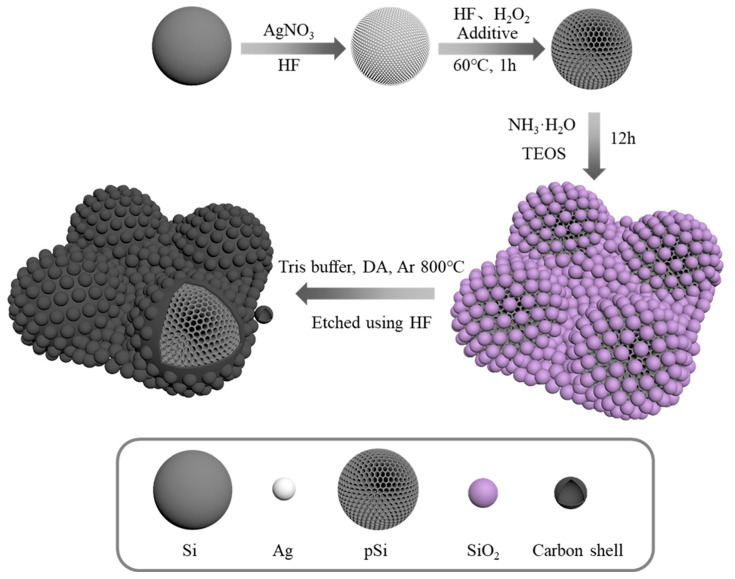
Schematic illustration of formation procedure of the p-Si, the p-Si@SiO_2_ and the p-Si@C composites.

Silver-assisted chemical etching is a redox process. As shown in Figure 1, firstly, the Si powder is silver plated, Ag^+^ trapped electrons from the valence band of Si and adhered to the Si particles, and Si lost electrons and was oxidized to SiO_2_, which HF dissolved [45]. After that, the Si powder deposited with silver nanoparticles was immersed in an etching solution of H_2_O_2_ and HF, and silver could be used as a catalyst to make the H_2_O_2_ preferentially reduced on the surface of silver. The holes generated by the reduced H_2_O_2_ were injected into the Si contacted with silver, and Si lost electrons and was oxidized to SiO_2_, which HF dissolved. The presence of H_2_O_2_ makes the reaction more intense [39]. In addition, the formation of holes is also related to the molar ratio of the etching solution; when the molar ratio is between 70% and 100%, it can form straight or curved cylindrical holes [46]. After exploration, an etchant with a molar ratio of about 80% was finally chosen.

The as-prepared p-Si@C composites contained carbon shell interconnections, SiO_2_ nanospheres as templates, and PDA as a carbon source. TEOS underwent hydrolysis via the sol–gel method, forming SiO_2_ nanospheres. Some of these nanospheres adhere to the p-Si particles, while others occupy the interstitial spaces between them. Afterwards, the p-Si@SiO_2_ mixtures and DA were added to a Tris buffer solution to help with the polymerization of DA on the surface of the p-Si@SiO_2_ mixtures. The resulting materials underwent annealing at 800 °C for 3 h under an Ar atmosphere, forming p-Si@SiO_2_@C composites. Finally, the SiO_2_ coating was eliminated using HF etching, leading to the creation of p-Si@C compounds connected by conductive carbon shell structures.

**Figure 2:**
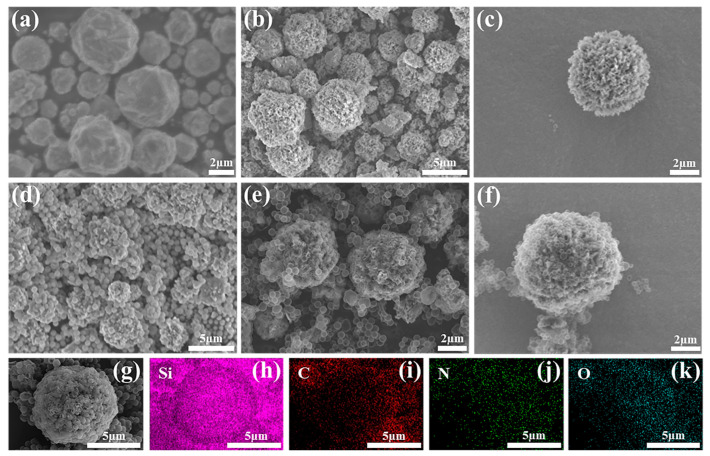
SEM images of the Si (**a**), the p-Si (**b**,**c**), the p-Si@SiO_2_ (**d**) and the p-Si@C composite (**e**,**f**) and the corresponding elemental mapping images of Si (**h**), C (**i**), N (**j**) and O (**k**) of the p-Si@C (**g**) composites.

SEM images of raw Si particles, p-Si, p-Si@SiO_2_, and p-Si@C composites, along with the corresponding EDS spectra, are shown in Figure 2. In Figure 2a, the average diameter of raw Si particles is approximately 3–5 μm, and the porous Si particles exhibit rich pore structures on the surface, which was achieved through a low concentration of silver nitrate (Figure 2b,c). The SiO_2_ nanoparticles not only covered the p-Si particles but also fill the gaps between them, as depicted in Figure 2d. Additionally, Figure 2e,f present the establishment of a conductive network interconnected by hollow carbon nanospheres after the pyrolysis of PDA and the subsequent SiO_2_ etching. The EDS image in Figure 2 shows that elements such as C, N, and O are uniformly distributed on Si.

**Figure 3:**
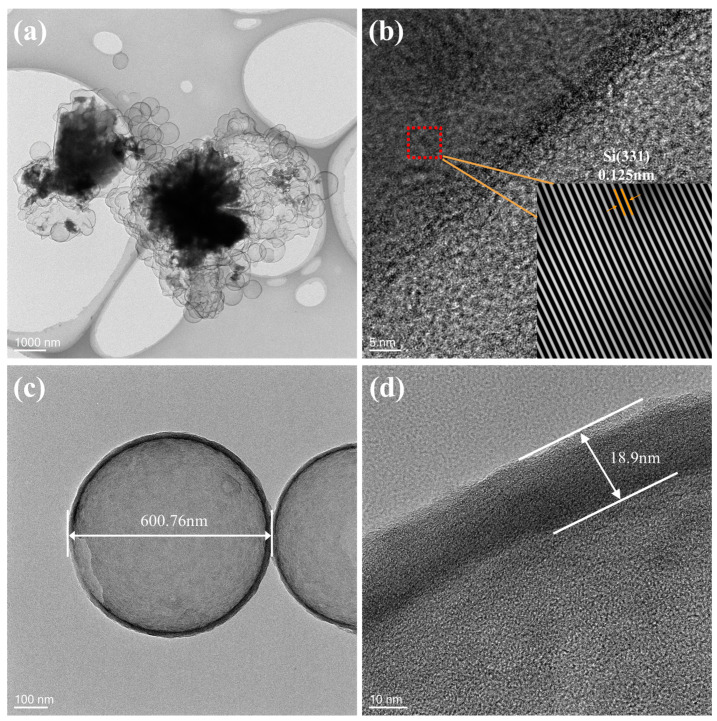
Low-magnification (**a**) and high-magnification (**b**) TEM image of the p-Si@C composites, (**c**,**d**) hollow carbon nanospheres and their wall thicknesses.

The structure of the as-prepared p-Si@C composites was additionally examined via TEM analysis. The interconnected network structure is distinctly visible in Figure 3a, where internal shadow particles, representing the p-Si particles, are effectively enveloped by nanosphere originating from the hollow carbon shell. In Figure 3b, the high-resolution TEM (HRTEM) image of the composite reveals a crystal plane spacing of approximately 0.125 nm, which is consistent with the (331) crystal plane of Si. This indicates that the p-Si particles are surrounded by hollow carbon spheres. Figure 3c,d also confirm the successful as-prepared hollow carbon nanospheres with the size of ~600.76 nm and ~18.9 nm wall thicknesses.

**Figure 4:**
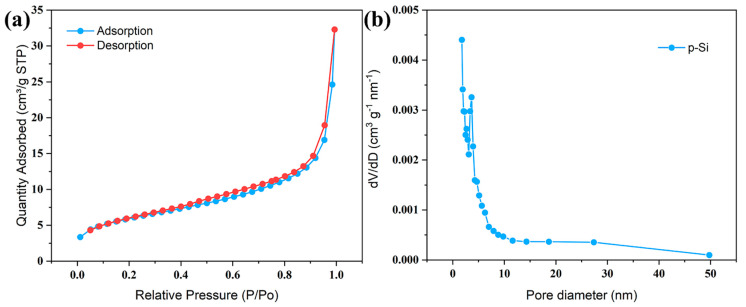
N_2_ adsorption–desorption isotherms; (**a**,**b**) BJH mesopore size distributions of the p-Si.

The pore size distribution and specific surface area of the p-Si were determined using BET analysis. As depicted in Figure 4a, the BET surface area of the p-Si is 21.45 m^2^·g^−1^. Furthermore, the N_2_ adsorption–desorption isotherms reveal that the p-Si exhibits a type-IV curve with H4 hysteresis loops, indicating the presence of both mesoporous and microporous structures [47,48], which is further supported by the pore size distribution curve in Figure 4b, where the average pore size of the p-Si is determined to be 11.56 nm.

**Figure 5:**
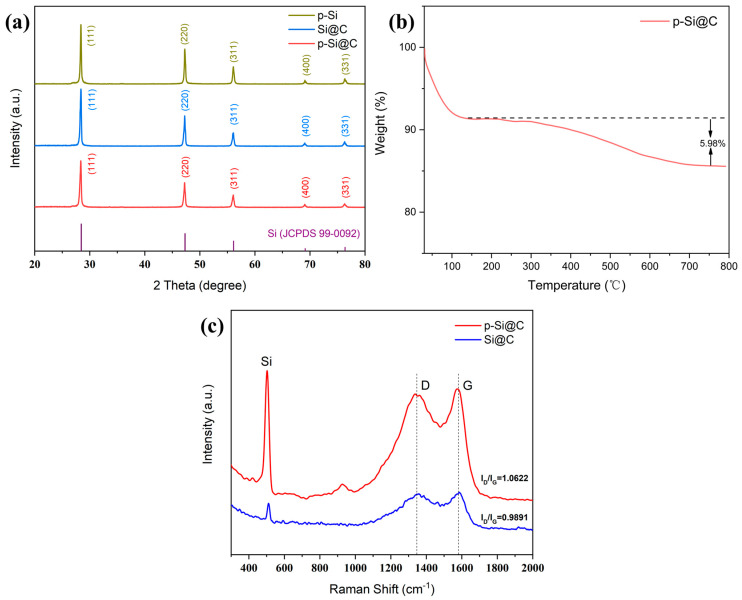
(**a**) XRD patterns of p-Si, Si@C and p-Si@C composites; (**b**) TG curves of p-Si@C composites; (**c**) Raman spectra of the Si@C and p-Si@C composites.

Figure 5a shows XRD patterns for Si@C, p-Si@C composites, and p-Si particles. The materials show diffraction patterns with five peaks at 28.4, 47.3, 56.1, 69.1, and 76.3, representing crystal planes (111), (220), (311), (400), and (331) of Si (JCPDS 99-0092), suggesting the crystal structure remained intact during preparation. Figure 5b shows the thermogravimetric profiles of the p-Si@C blends acquired under air conditions ranging from 30 to 800 °C. The loss of weight at temperatures below 120 °C is usually caused by the evaporation of water that is adsorbed and water that is part of the crystalline structure [49]. In contrast, the more rapid reduction in weight after 180 °C is attributed to the carbonation. The carbon mass fraction is around 5.98%. Figure 5c displays the Raman spectra of the p-Si@C and Si@C composites. Two spectral curves exhibit three peaks at around 510, 1340, and 1590 cm^−1^, corresponding to Si and the D and G bands of carbon. The p-Si@C composites had an ID/IG ratio of 1.0622, while the Si@C composites had a ratio of 0.9891, suggesting that the amorphous carbons in the hollow carbon shell of the composites are responsible for this difference [50].

**Figure 6:**
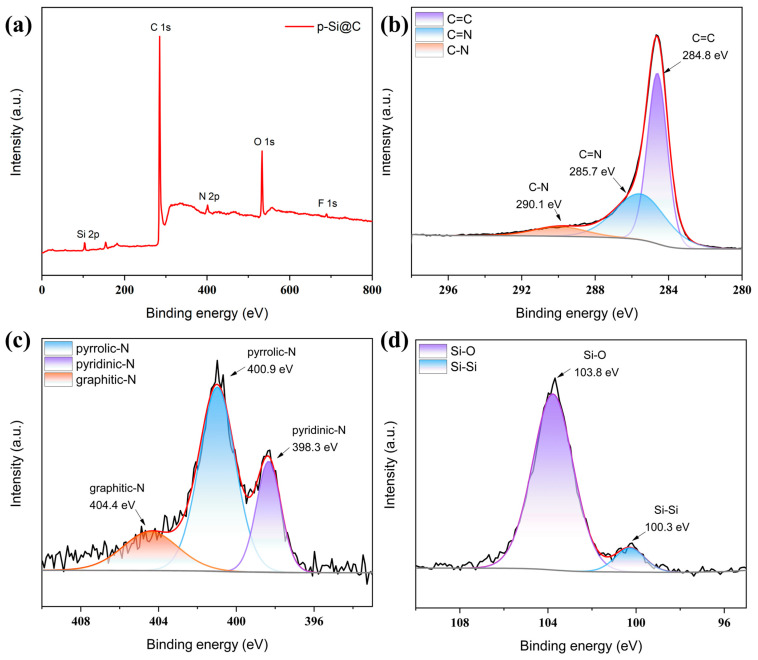
(**a**) XPS survey of the p-Si@C composites; the high-resolution XPS spectra of (**b**) C 1 s, (**c**) N 1 s, and (**d**) Si 2p of the p-Si@C composites.

The elemental composition of the p-Si@C composites was analyzed using XPS. The results are presented in Figure 6a, indicating the presence of Si, C, N, O, and F in the p-Si@C composites. The introduction of F is presumed to occur during the use of HF to etch the p-Si and SiO_2_ layers, the EDS spectra of p-Si@C composites are shown in Figure S1. The XPS spectra of C 1s are shown in Figure 6b, where the peaks at 284.8 eV, 285.7 eV, and 290.1 eV are assigned to the C=C, C=N, and C-N bonds [51]. The presence of a C=C double bond may be due to the condensation of phenolic functional groups to form an aromatic ring structure during the pyrolysis of PDA, which generates π-π bonding interactions to form a conductive network. The presence of C-N bonds indicates the existence of N-doped carbon, which is primarily resulting from the pyrolyzed PDA [52]. In Figure 6c, three peaks of the N element correspond to pyridinic-N (398.3 eV), pyrrolic-N (400.9 eV), and graphitic-N (404.4 eV), respectively [53]. Among them, the structure of graphitic-N is similar to graphite, which facilitates electron conduction and enhances electrode conductivity [54]. Figure 6d illustrates two peaks related to Si with the peak at 100.3. eV attributed to Si-Si and the peak at 103.8 eV attributed to Si-O. The Si-O bond indicates the presence of SiO_X_, which is possibly resulting from residue while etching the SiO_2_.

**Figure 7:**
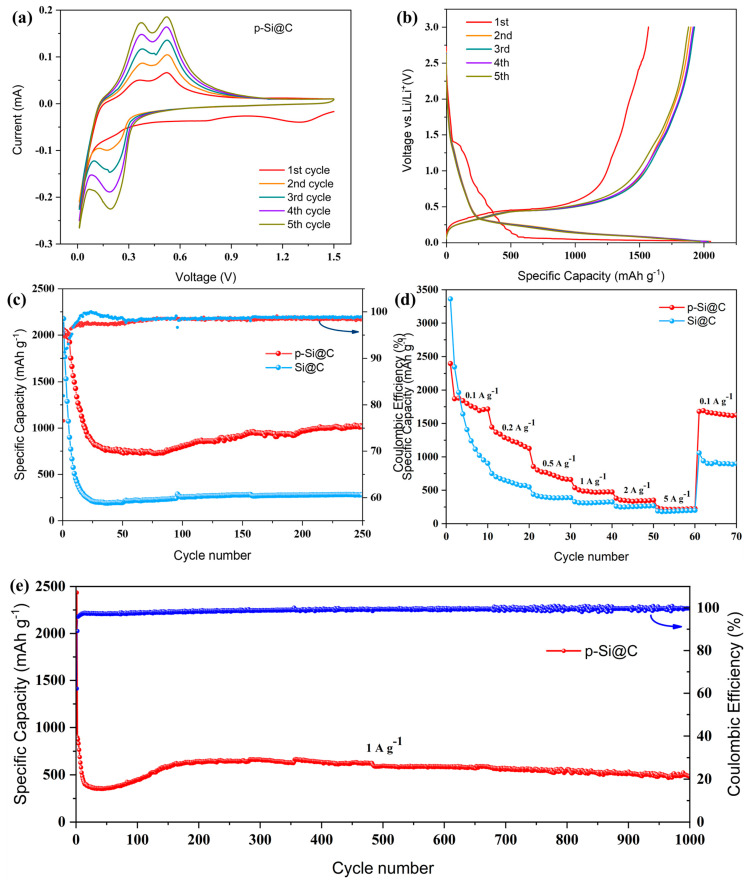
(**a**) CV tests of the p-Si@C composites for the first five cycles; (**b**) the charge–discharge curves for the first five cycles, (**c**) cycling performance at 0.2 A·g^−1^ and (**d**) rate performance at various current densities for the Si@C and the p-Si@C composites; (**e**) long-term cycle of the p-Si@C composites at 1 A·g^−1^.

Testing was conducted on the electrochemical characteristics of the p-Si@C composites. In Figure 7a, the cyclic voltammetry (CV) profile, conducted in the voltage range of 0.01–1.50 V (vs. Li/Li^+^), is presented for the initial five cycles at a scan rate of 0.10 mV·s^−1^. The presence of a cathode peak at 0.72 V during the first cycle may suggest the development of the SEI film on the active material’s surface, which could lead to an irreversible loss of capacity [55]. The subsequent cycles show the absence of the cathode peak, indicating the stability of the SEI layer formed on the surface of the active material. This stability can be primarily attributed to the porous structure of the p-Si, which alleviates the mechanical stresses arising from volume expansion and prevents the breakage of the SEI film. Notably, a cathode peak at 0.17 V and two anode peaks around 0.32 V and 0.51 V are observed, corresponding to lithium-ion insertion/deinsertion [56,57], the CV tests of Si@C composites are shown in Figure S2. Figure 7b depicts the charge–discharge voltage curves at a current density of 0.2 A·g^−1^. The extended and flat plateau observed around 0.1 V during the initial lithiation process indicates the alloying process of the composites. The charge–discharge curves of the electrodes for the subsequent four cycles overlapped almost exactly, indicating that the p-Si@C composites have reversible electrochemical properties. Figure 7c demonstrates the cycling performance testing of the composites. In a control experiment, the Si@C composites demonstrate an initial discharge specific capacity of 2176.74 mAh·g^−1^ with an initial coulombic efficiency of 81.91%. After 250 cycles, a reversible capacity of about 275.09 mAh·g^−1^ is obtained. In contrast, the p-Si@C composites demonstrate an initial discharge specific capacity of 2056.22 mAh·g^−1^ with an initial coulombic efficiency of 76.51%. The enhanced surface area stemming from the porous structure is responsible for the lower initial coulombic efficiency observed in the p-Si@C composites, as it fosters additional irreversible reactions with the electrolyte. After 250 cycles, the composites exhibit a sustained capacity of 1008.84 mAh·g^−1^. The rapid capacity degradation observed is ascribed to the porous structure, which enhances the surface area of the active material and facilitates the formation of an SEI film within the pores. Upon cell activation, the capacity of the electrodes shows an increasing trend, which was followed by a gradual stabilization. Figure 7d presents the rate performance of the p-Si@C and Si@C composites at various current densities ranging from 0.1 to 5 A·g^−1^. The p-Si@C composite delivers discharge capacities of 2236.92, 1447.64, 857.97, 539.70, 380.85, and 237.66 mAh·g^−1^. Upon returning to a current density of 0.1 A·g^−1^, the specific capacity of the p-Si@C composites reaches 1683.6 mAh·g^−1^. Furthermore, as illustrated in Figure 7e, under the long cycle test with high current, the composite still has stable reversible cycling capacity, and the discharge-specific capacity of the 1000th cycle reaches 485.93 mAh·g^−1^, which indicates that the SEI film has not been damaged by the composite material. The exceptional reversibility of the composites at high current densities is primarily due to the porous structure and the carbon layer, both of which contribute to enhanced electrode stability. In contrast, the reversibility of Si@C composites is poor. When the current density was reduced from 5 to 0.1 A·g^−1^, the specific capacity of Si@C composites reached only 1060.88 mAh·g^−1^, which further confirmed the positive effect of p-Si on mitigating the volume expansion.

**Figure 8:**
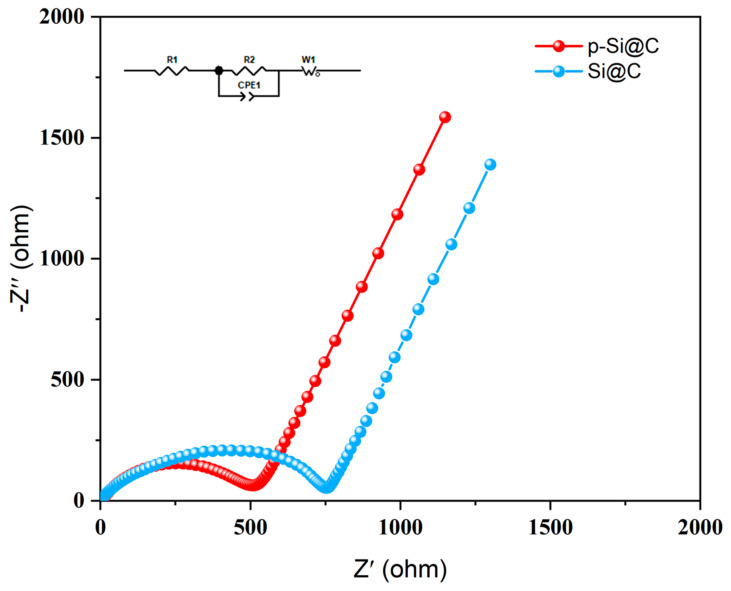
EIS of the p-Si@C composites before charge/discharge cycling.

Figure 8 illustrates the EIS test for composites, where the curve consists of two components: the linear part of the low-frequency region corresponds to the Li^+^ diffusion limitation process, and the semicircular portion of the high-frequency region corresponds to the charge transfer-limiting process [58]. The smaller diameter of the semicircle for the p-Si@C composites indicates a lower charge transfer resistance. The p-Si@C composites exhibit a charge transfer resistance of 501.94 ohms, while the Si@C composites show a resistance of 748.87 ohms. This difference may stem from the increased surface area of p-Si, which shortens the distance for charge transport.

**Figure 9:**
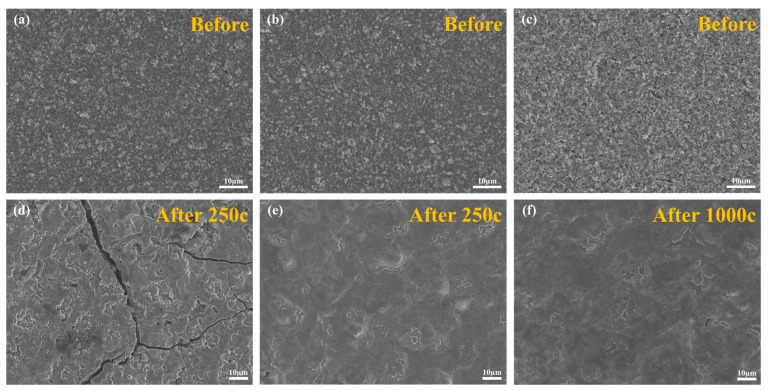
The SEM images of Si@C electrode before (**a**) and after cycles (**d**) and p-Si@C electrode before (**b**,**c**) and after cycles (**e**,**f**).

Figure 9 presents an SEM image of the electrode captured using a non-in situ method. In Figure 9a,d, the initial appearance of the Si@C electrode is displayed as well as the structure after undergoing 250 cycles at a current density of 0.2 A·g^−1^. Extensive cracking in the electrode is the result of the significant volume expansion experienced by pure Si. In contrast, the p-Si@C electrode did not crack after 250 cycles and remained unchanged significantly even after 1000 cycles at a current density of 1 A·g^−1^. The notable disparity between the two electrodes suggests that the p-Si plays a positive role in mitigating the volume expansion of Si and prevents electrode rupture.

## 4. Conclusions

In conclusion, micron-sized p-Si was successfully synthesized at a low silver ion concentration and exhibited excellent cycling stability in the p-Si@C electrodes. This enhanced performance can be credited to the porous structure and the hollow carbon nanospheres, which is a special structural design that effectively improves the electrochemical performance of the electrodes. p-Si’s porous structure and the hollow carbon nanospheres absorb the mechanical stresses generated by the volume expansion, which effectively mitigates the volume expansion problem and prevents the electrodes from cracking. Electrochemical assessments demonstrate that the p-Si@C composites display remarkable cycling stability and rate capability. Specifically, the composites demonstrate a noteworthy reversible specific capacity of 1008.84 mAh·g^−1^ after 250 cycles under a current density of 0.2 A·g^−1^ and maintain a reversible specific capacity of 237.66 mAh·g^−1^ under a current density of 5 A·g^−1^. In prolonged cycling tests, the p-Si@C composites sustain a discharge capacity of 485.93 mAh·g^−1^ after 1000 cycles at 1 A·g^−1^. Preparation of the p-Si with a rich porous structure using silver-assisted chemical etching holds promise for next-generation lithium-ion battery anodes.

## Data Availability

The original contributions presented in the study are included in the article, further inquiries can be directed to the corresponding author.

## Supplementary Materials

The following supporting information can be downloaded at: https://www.mdpi.com/article/10.3390/ma17133189/s1, Figure S1. EDS spectra of p-Si@C. Figure S2. CV tests of the Si@C composites for the first five cycles.
