# Supplementary material for: Silver-Assisted Chemical Etching for the Fabrication of Porous Silicon N-Doped Nanohollow Carbon Spheres Composite Anodes to Enhance Electrochemical Performance
Source: Materials (Basel). 2024 Jun 29;17(13):3189. doi: 10.3390/ma17133189 (PMC11242775; doi:10.3390/ma17133189)
Supplement: Supplementary file 1 [file materials-17-03189-s001.zip › materials-3039981-supplementary.pdf]

Article

Silver-Assisted Chemical Etching for the Fabrication of Porous Silicon N-Doped Nanohollow Carbon Spheres Composite Anodes to Enhance Electrochemical Performance

Zimu Zhang, Yuqi Zhang, Weixuan Chen, Xiang Zhang, Le Yu and Zisheng Guan *

College of Materials Science and Engineering, Nanjing Tech University, 30 South PuZhu Road, Nanjing 210009, China; 202161203162@njtech.edu.cn (Z.Z.); 202161203205@njtech.edu.cn (Y.Z.); 202161203185@njtech.edu.cn (W.C.); 202161203285@njtech.edu.cn (X.Z.); 202261203158@njtech.edu.cn (L.Y.)

* Correspondence: zsguan@njtech.edu.cn; Tel.: +86-138-1393-2186

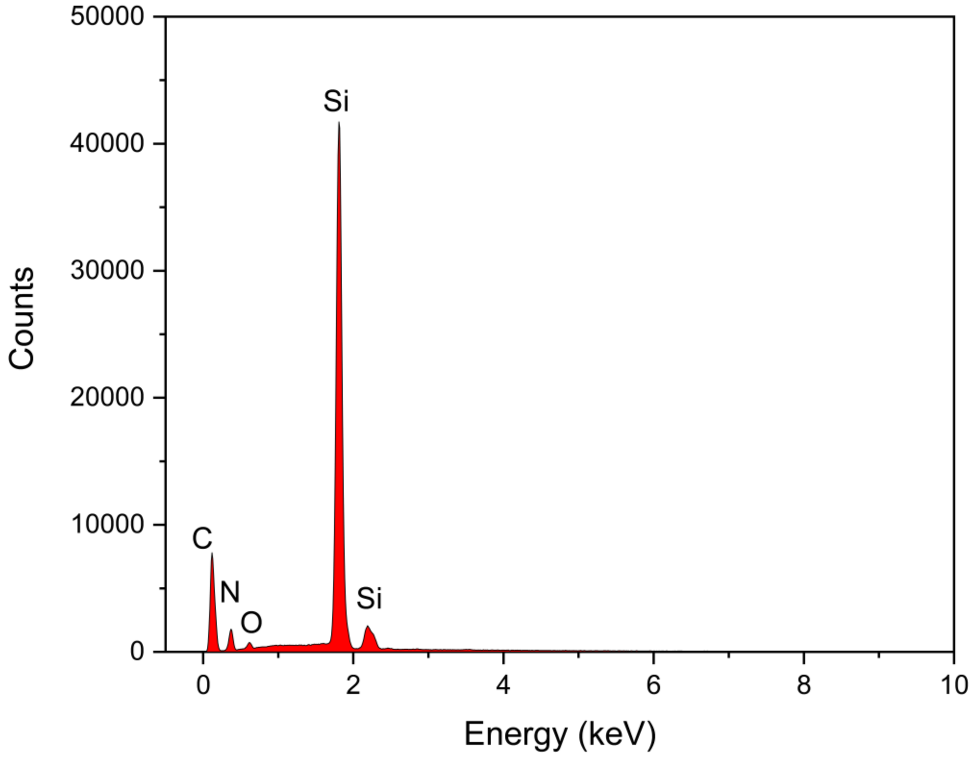

Figure S1. EDS spectra of p-Si@C.

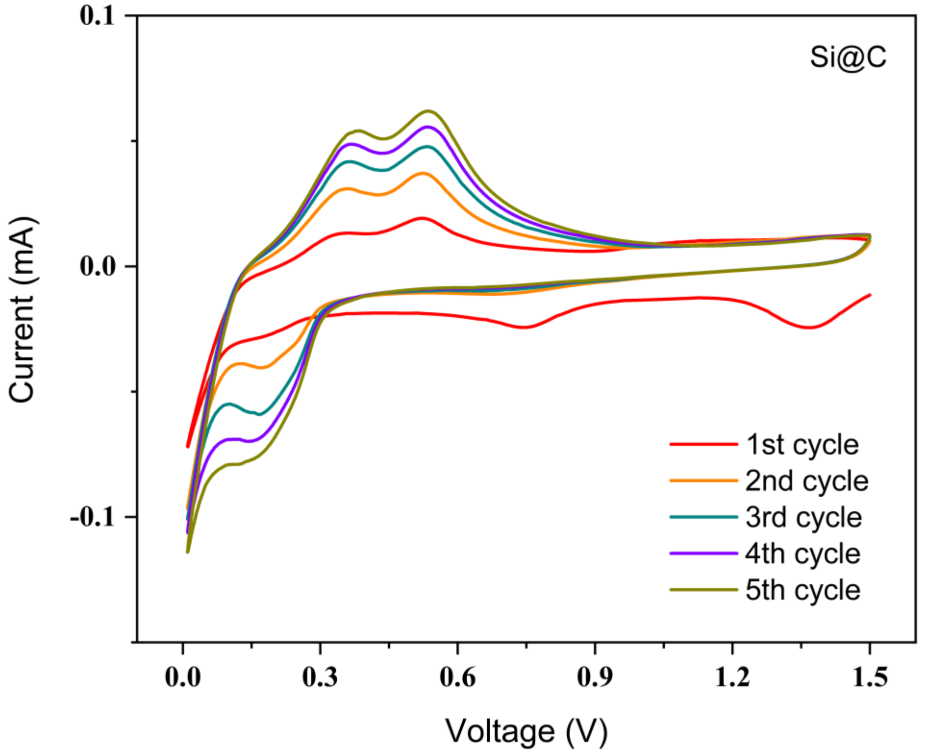

Figure S2. CV tests of the Si@C composites for the first five cycles.
